# Molecular Drivers of Pancreatic Cancer Pathogenesis: Looking Inward to Move Forward

**DOI:** 10.3390/ijms18040779

**Published:** 2017-04-06

**Authors:** Mohammad Aslam Khan, Shafquat Azim, Haseeb Zubair, Arun Bhardwaj, Girijesh Kumar Patel, Moh’d Khushman, Seema Singh, Ajay Pratap Singh

**Affiliations:** 1 Department of Oncologic Sciences, Mitchell Cancer Institute, University of South Alabama, Mobile, AL 36604, USA; makhan@health.southalabama.edu (M.A.K.); sazim@health.southalabama.edu (S.A.); hzubair@health.southalabama.edu (H.Z.); abhardwaj@health.southalabama.edu (A.B.); gpatel@health.southalabama.edu (G.K.P.); seemasingh@health.southalabama.edu (S.S.); 2 Departments of Interdisciplinary Clinical Oncology, Mitchell Cancer Institute, University of South Alabama, Mobile, AL 36604, USA; mmkhushman@health.southalabama.edu; 3 Department of Biochemistry and Molecular Biology, College of Medicine, University of South Alabama, Mobile, AL 36604, USA

**Keywords:** pancreatic ductal adenocarcinoma, molecular pathogenesis, tumor microenvironment, non-coding RNAs, mutations, microRNA

## Abstract

Pancreatic cancer (PC) continues to rank among the most lethal cancers. The consistent increase in incidence and mortality has made it the seventh leading cause of cancer-associated deaths globally and the third in the United States. The biggest challenge in combating PC is our insufficient understanding of the molecular mechanism(s) underlying its complex biology. Studies during the last several years have helped identify several putative factors and events, both genetic and epigenetic, as well as some deregulated signaling pathways, with implications in PC onset and progression. In this review article, we make an effort to summarize our current understanding of molecular and cellular events involved in the pathogenesis of pancreatic malignancy. Specifically, we provide up-to-date information on the genetic and epigenetic changes that occur during the initiation and progression of PC and their functional involvement in the pathogenic processes. We also discuss the impact of the tumor microenvironment on the molecular landscape of PC and its role in aggressive disease progression. It is envisioned that a better understanding of these molecular factors and the mechanisms of their actions can help unravel novel diagnostic and prognostic biomarkers and can also be exploited for future targeted therapies.

## 1. Introduction

Pancreatic cancer (PC) is the seventh most common cause of cancer-associated deaths around the world, and 418,000 new cases of PC are estimated to be diagnosed worldwide in 2020. The incidence and mortality rates of PC are particularly high in the developed countries [1,2]. In the United States, PC is the third leading cause of cancer-related deaths. According to the American Cancer Society, approximately 53,670 people will be diagnosed with PC and nearly 43,090 will die from this disease in 2017 [3]. The median overall survival for PC patients is 2–8 months, and five-year survival is 7.7% [3]. A major factor contributing to this dismal prognosis of PC is its asymptomatic progression. PC is mostly diagnosed at a stage when it has already metastasized or is locally advanced, thus limiting the potential for therapeutic intervention [4,5,6]. No reliable biomarkers are currently available that could help in the early detection of PC. Sialyl Lewis (carbohydrate antigen 19-9 (CA19-9)) is a carbohydrate antigen that serves as a ligand of selectin, a widely used serum biomarker for PC [7], which has limitations related to its sensitivity and specificity. CA19-9 is expressed only in individuals with Lewis a+/b− or Lewis a+/b+ genotypes. Moreover, elevated levels of CA19-9 are also detected in some non-cancerous conditions, as well, including pancreatitis [7]. Over the past several years, many new candidate RNA and protein biomarkers have been identified for the early diagnosis of PC, yet their sensitivity and specificity remain to be tested in larger patient cohorts. Moreover, in recent years, novel sources of biomarker detection, such as circulating tumor cells and exosomes, have also been explored [8]. This progress is promising; however, it remains far from reaching clinics for screening or diagnostic use. 

A better understanding of the molecular pathogenesis of PC can be immensely helpful in the development of novel biomarkers and effective therapeutic strategies. Research progress in recent years has helped identify several molecular alterations that occur during its progression [9,10,11]. It is believed that the initiation and progression of PC are associated with successive accumulation of alterations in multiple genes critical for tumorigenesis, leading to deregulation of several oncogenic signaling pathways [12,13,14,15,16]. This review comprehensively details our current understanding of these aspects and discusses the potential that this knowledge holds for the development of effective PC management strategies.

## 2. Clinical Progression of Pancreatic Cancer

**Figure 1:**
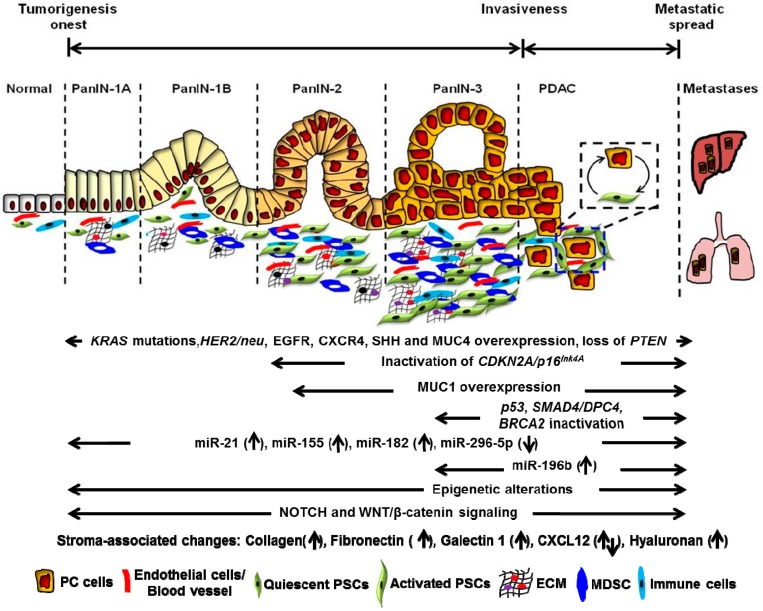
Histopathological and molecular changes in the pathogenesis of pancreatic adenocarcinoma (PDAC). The illustration describes the multistep PDAC development, starting from normal epithelium to low-grade pancreatic intraepithelial neoplasia (PanINs) and on to high-grade PanIN and invasive carcinoma. During this progression, several alterations in key genes (*KRAS*, *CDKN2*, *TP53*, *SMAD4/DPC4* and *BRCA2*) are accumulated. Apart from genetic alterations, deregulated signaling pathways, stromal associated factors and microRNAs serve as fuel for the development of aggressive pancreatic cancer. KRAS: Kirsten rat sarcoma oncogene homolog; Her-2/neu: Human epidermal growth factor receptor 2; EGFR: Epidermal growth factor receptor; CXCR4: C-X-C chemokine receptor type 4; SHH: Sonic hedgehog; MUC4: Mucin 4; PTEN: Phosphatase and tensin homolog; CDKN2A/p16^Ink4A^: Cyclin dependent kinase inhibitor 2A; SMAD4/DPC4: Mothers against decapentaplegic homolog 4/ Deleted in pancreatic cancer-4; BRCA2: Breast cancer type 2 susceptibility protein; CXCL12: C-X-C motif chemokine 12.

Development and progression of PC is a multistep process (Figure 1). Pancreatic cancer progresses from noncancerous precursor lesions called pancreatic intraepithelial neoplasia (PanIN) lesions to invasive carcinoma [12,17]. Based on the degree of cellular and nuclear atypia, PanIN lesions are further sub-divided into low- (PanIN-1A/B) to high- (PanIN-3) grade lesions [18]. As shown in Figure 1, numerous genetic changes accumulate over time and drive histologic progression through the PanIN stages (PanIN1–3), ultimately leading to invasive adenocarcinoma. These changes include genetic mutations, gain or loss of various tumor promoting/suppressor genes and microRNAs (miRNAs) [16,17,19,20]. In early low-grade PanIN lesions (PanIN-1), Kirsten rat sarcoma oncogene homolog (*KRAS)* is mutated, oncogenic miRNAs are overexpressed and stromal associated factors are activated. Mucin 1 (*MUC1*) is overexpressed, and inactivating mutations in the *p16/CDKN2A* gene are observed in intermediate lesions (PanIN-2). Finally, late lesions (PanIN-3) are associated with inactivating mutations in tumor protein p53 (*TP53*), breast cancer type 2 susceptibility protein (*BRCA2*) and mothers against decapentaplegic homolog 4 (*SMAD4*). With the growing interest in identifying the causal factors responsible for the development and progression of PC [13,16,21,22], phosphatidylinositol-3,4,5-trisphosphate-dependent rac exchange factor 2 (*PREX2*) and lysine demethylase 6A (*KDM6A*) have recently been identified as two new drivers of pancreatic tumorigenesis [23].

The tumor microenvironment (TME) also plays an important role in PC pathogenesis and the failure of therapeutic intervention. Pancreatic tumors are not only composed of tumor cells, but are marked by several other cell populations, such as fibroblasts, immune cells and endothelial cells. Growing evidence suggests that tumor-stromal interactions play a major role in tumorigenesis, both at primary and secondary sites [11,24,25]. Tumor cells remodel the surrounding stroma during the course of malignant progression and develop a reciprocal association with surrounding stroma to cooperatively promote their own growth [26]. Stroma-derived cytokine CXCL12 is taken up by tumor cells and promotes tumor progression, metastasis and chemoresistance. Pancreatic tumor cells produce sonic hedgehog (SHH), which induces desmoplasia in a paracrine mechanism and supports pancreatic tumorigenesis [26,27]. Earlier studies proclaimed the tumor supportive role of stroma in PC pathogenesis. However, a contrasting role of stroma has also been reported wherein depletion of stroma triggers immune suppression and collectively worsens the disease [28,29]. The dual properties of tumor stroma open a new area to explore the role of the tumor microenvironment in the pathogenesis of pancreatic adenocarcinoma (PDAC). The question as to how stroma is regulated in the TME to support tumor growth remains largely unanswered. Unlike tumor cells, genetic alterations are very rare in the stromal cells, and it may be possible that epigenetic alterations influence the phenotype of fibroblasts [30,31]. This notion is supported by current findings that pancreatic tumor cells are able to induce DNA methylation of the suppressor of cytokine signaling 1 (SOCS1) gene in cancer-associated fibroblasts (CAFs), and these epigenetically-regulated CAFs potentially promote the growth of tumor cells in vivo [32]. Further, a study of metabolic interactions suggests that pancreatic tumor cells use stromal cells (pancreatic stellate cells) as energy reservoirs to fulfill their energy demands [33]. It has also been suggested that KRAS-mediated oncogenic signaling requires pancreatic stroma to support pathogenesis. Furthermore, this study suggested that the stromal signal induces distinct gene patterns in PC cells, an observation that is supported by increased histone acetylation, an epigenetic alteration, suggesting the importance of stroma in reprogramming PC cells [34]. 

In PC, excessive desmoplastic reaction induces the proliferation of fibroblast cells and the production of extracellular matrix (ECM). Extracellular matrix, the non-cellular component, is made up of collagens, elastins, fibronectins, osteonectin, laminins and hyaluronan. Extracellular matrix forms fibrous mesh around tumor cells, and it has recently been shown that hyaluronan (HA), a major component of ECM, provides favorable microenvironment for PC progression by promoting malignant cell proliferation, aggressiveness and resistance to cancer therapy, resulting in poor prognosis [35,36]. Recent pre-clinical findings suggested that enzymatic inhibition of HA by hyaluronidase, in combination with gemcitabine, can significantly increase the overall survival of tumor-bearing mice [37].

Pancreatic tumors are highly metastatic, and in a majority of PC patients, tumors have already metastasized at the time of detection. Pre-metastatic niche formation is recognized as one of the reasons for the early spread of tumor cells. It has been shown that tumor cell-derived tissue inhibitor of metalloproteinases-1 (TIMP1) plays a role in liver metastasis of PC [38]. Communication between cancer cells, as well as between cancer cells and other cells, though membrane-bound vesicles (exosomes), is also involved in the early metastases of pancreatic cancer. Exosomes are now widely accepted as cellular messengers, found to be involved in many pathological conditions, including cancer [39]. The levels of macrophage migration inhibitory factor (MIF)-positive exosomes were relatively higher in the PDAC patients who eventually developed liver metastases, as compared to non-metastatic patients [40]. Furthermore, these MIF-positive exosomes, derived from tumor cells, help form a pre-metastatic niche, establishing the tumor at a secondary site, liver [40]. Recently, it has been shown that fibroblast-secreted exosomes help in the metabolic reprogramming of tumor cells by providing metabolite cargo, promoting the growth of tumor cells during starvation or other stress conditions [41]. We have also shown that chemotherapy-induced exosomes from pancreatic tumor cells impart chemoresistance in PC [42]. Additionally, it has been suggested that exosomes from other sources, such as stroma, can support PC growth and survival [43]. Another study suggested that pancreatic tumor cell-derived exosomes have certain integrins, and the patterns of these integrins determine the organotrophic metastasis of tumor cells [44]. Moreover, it was suggested that glypican-1 (GPC1)-positive exosomes, derived from cancer cells, may potentially serve as a diagnostic tool to detect early stages of pancreatic tumor [45], and in a follow-up study, the same group suggested that circulating exosomes could be efficiently used for the identification of cancer-driving mutations in PC [46]. All of these findings suggest that exosomes can be used as diagnostic/prognostic markers and also act as one of the key components of chemoresistance in pancreatic cancer.

It is thus clear that PC pathogenesis involves a complex interplay of multiple factors. In addition to the different interactions of tumor cells with their immediate surroundings, as discussed above, several molecular alterations are responsible for PC onset and progression. In the section below, we discuss the current knowledge on genetic alterations found to be associated with PC pathogenesis.

## 3. Molecular Alterations Driving Pancreatic Cancer Progression

Several molecular events are now believed responsible for the progression of PC. These include genetic, as well as epigenetic changes. Genetic events include mutations in key genes, activation of oncogenes and inactivation of tumor suppressors. In addition, epigenetic regulation through non-coding RNAs is also increasingly being recognized in PC initiation, as well as progression (Figure 1). This section summarizes many of these reported molecular alterations. 

### 3.1. Activation of Oncogenes

Oncogenic *KRAS* has been extensively studied in PC because of the high prevalence of its mutations. *KRAS* mutations are detected in ~30% of early neoplasms with the frequency rising to ~95% in advanced PC [47,48]. The activating mutations of *KRAS* are the point mutations at codon G12 (GGT to GAT/GTT/CGT) resulting in the substitution of glycine with aspartate, valine or arginine [47]. These mutations at codon 12 represent the major point mutations (~98%) with some mutations reported at codons 13 and 61, as well [47]. Mutated *KRAS* results in the constitutive activation of its downstream oncogenic signaling [47,49]. It has been suggested that oncogenic *KRAS* signaling is required for initiation, progression and maintenance of PC [50]. Moreover, it has been reported that constitutive activation of *KRAS* results in the activation of pancreatic stellate cells and immune cells, thus further contributing to the neoplastic progression of PC [49]. Although a critical role of KRAS protein has been identified in PC, this has not resulted in any therapeutic advantage, as KRAS remains a non-druggable target [48]. This realization has resulted in efforts to develop therapies that target signaling upstream or downstream of KRAS [48,51,52]. In pre-clinical mouse models, successful targeting of KRAS has been reported. For instance, through systemic administration of nanoparticles containing KRAS-specific small interfering RNA (siRNA), regression of KRAS-driven tumors was observed [52,53]. Several drugs targeting rapidly accelerated fibrosarcoma (RAF)-mitogen-activated protein kinase (MAPK) and phosphatidylinositide 3-kinase (PI3 kinase), the well-established downstream pathways of KRAS, are being tested in the clinics [54].

c-MYC proto-oncogene is an important regulator of many cellular functions in normal and cancer cells [55]. It has been shown to affect the expression of proteins facilitating cell transformation, cell growth, cell cycle progression, cellular stemness and cellular metabolism [55,56,57]. The alteration of c-MYC alone is not sufficient for the development of pancreatic tumors [58]. However, concurrent alteration of c-MYC expression and mutation in the *KRAS* gene is sufficient to instigate tumorigenesis [58]. Similarly, c-MYC overexpression and exposure to TGFα results in the development of pancreatic acinar lesions in the mouse model, as opposed to the absence of tumor with either of the two conditions alone [59]. The gene encoding c-MYC on chromosome 8q is reported to be amplified in 20–30% of PC cases [60]. The nuclear factors of activated T cells (NFAT) family of transcription factors has been found to be overexpressed in pancreatic tumors and is responsible for increased c-MYC gene expression [61]. Increased p300-dependent histone acetylase activity, after binding of NFAT to the c-MYC promoter, has been demonstrated to enhance the binding of other factors to promote fully active c-MYC transcription [61]. On the other hand, enhanced c-MYC protein stability by NAD-dependent deacetylase sirtuin-2 (SIRT2) or inhibitor of nuclear factor kappa B kinase subunit epsilon (IKKε) has been demonstrated to increase pancreatic cancer tumorigenicity [10,62]. 

The p21-activated kinase 4 (*PAK4*) gene is amplified on the chromosome19q13, a region found frequently amplified in pancreatic cancer [63]. In addition to its amplification, PAK4 has been shown to be overexpressed in various tumors types, including PC [14,63,64]. Recently, we have demonstrated that PAK4 is overexpressed in PC, and it promotes the proliferation and survival of PC cells through AKT (RAC-alpha serine/threonine-protein kinase)- and ERK (extracellular signal-regulated kinase)-dependent activation of the nuclear factor-kappa B (NF-κB) pathway [14]. In a follow-up study, we have also reported a role of PAK4 signaling in the induction of stemness and drug resistance in PC [65]. In addition, others have reported the role of PAK4 in enhancing the motility potential of PC cells [66].

The *MYB* proto-oncogene, a cellular progenitor of the *v-MYB* oncogenes carried by the chicken retroviruses *AMV* (*avian myeloblastsis virus*) and *E26*, encodes for the MYB transcription factor, which engages in gene regulation by binding to responsive promoter DNA sites [67]. MYB has been shown to induce the expression of many genes that regulate proliferation, differentiation and apoptosis. *MYB* is reported to be amplified in ~10% of pancreatic tumor samples, and interestingly, its amplification was predominantly reported in advanced tumors indicating a strong correlation with the progression and malignant properties of pancreatic adenocarcinoma tumors [68]. Similarly, we observed MYB to be overexpressed in a majority of PC patient-derived tissues and cell lines, with no expression detectable in normal pancreas. We, for the first time, reported a functional role of MYB in promoting the growth and aggressiveness of PC [69]. Our follow-up study identified that MYB potentially regulates the growth and genomic stability of PC cells by targeting complex gene networks and oncogenic signaling pathways [15]. We have also reported a critical role of MYB in pancreatic tumor histopathology and associated molecular and biological mechanisms. MYB-overexpressing tumors exhibit far-greater desmoplasia as compared to low MYB expressing/silenced tumors. Moreover, MYB-overexpressing PC cells confer significantly enhanced growth benefit to pancreatic stellate cells. Furthermore, we identified SHH and adrenomedullin (ADM) as the two molecular mediators responsible for MYB-induced desmoplasia [9]. Our observations from these studies are suggestive of MYB’s diverse roles in PC pathobiology.

Another oncogene, human epidermal growth factor receptor 2 (*HER2*), is also reported to be amplified in PC [70]. HER2 is a transmembrane growth factor receptor tyrosine kinase protein, which is encoded by the *ERBB2* gene located on human chromosome 17. HER2 is involved in the regulation of a wide range of cellular functions, including cell growth, survival and differentiation. Blocking of the HER2 receptor has been observed to improve survival in several cancers [71,72]. *HER2* amplification has been reported in ~2% of PC cases [73]. Moreover, *HER2* amplification positively correlates with lung and brain metastases [73].

### 3.2. Inactivation of Tumor Suppressor Genes

Cyclin-dependent kinase inhibitor 2A, also known as p16^INK4A^ or p16.p16^INK4A^, is a member of the Ink4 family of cyclin dependent kinase (CDK) inhibitors, which is involved in cell cycle regulation. p16 regulates G1/S phase cell cycle progression by binding to CDK4 and CDK6 to abolish their interaction with cyclin D1 [74]. Therefore, loss of function of p16 results in the progression of the cell cycle through the G1/S checkpoint in an unrestricted manner, leading to the enhanced cell proliferation [74,75]. The biological role and observed low expression of p16 make it a tumor suppressor protein in a majority of human malignancies [75]. Preclinical studies have suggested that tumor develops very rapidly in p16-knockout mice [76]. Inactivation of p16 is due to mutations or deletions. Mutations in the p16 gene result in a syndrome in humans, known as melanoma pancreatic cancer syndrome (MPCS) [77]. Genetic analyses have suggested that families with MPCS inherit melanoma susceptibility (20–40%), associated with mutations in *CDKN2A* located on chromosome 9p21. Individuals with this mutation are likely to be predisposed to pancreatic cancer [78]. Inactivation of p16 is first observed in moderately advanced early PanIN lesions (PanIN-1B). Moreover, the frequency of p16 inactivation is increased as PanIN-1B lesions progress to invasive carcinomas [79,80]. Loss of Ink4a has been reported to result in pancreatic neuroendocrine tumor (PanNET) in a mice model [81]. Forced expression of p16 inhibits pancreatic tumor growth in the orthotopic mice model and also inhibits lymph node metastases [82]. It has also been suggested that restoration of p16 inhibits PC cell proliferation [83]. Moreover, loss of p16 expression was observed in 67% of clinical samples representing lymphatic invasion and metastases of PC [84].

The *TP53* gene, located on chromosome 17p, is reported to be mutated in ~50–75% of PC patients. Studies suggest that intra-genic mutation, combined with a loss of the second allele, results in the inactivation of *TP53* gene. Inactivation of p53 leads to de-regulation of the cell cycle at G1-S and induction of apoptosis [85,86,87]. It has also been suggested that the p53 mutation is a late event in PC progression, generally observed in advanced PanIN lesions [88]. The altered p53 gene may give rise to a distinct pancreatic tumor morphology via coupling with other genetic abnormalities. This notion is supported by the observation that a mice model with one deleted copy of adenomatous polyposis coli (APC), a pancreatic developmental gene, along with p53 deletion, develops a distinct precursor of PDAC, mucinous cystic neoplasm (MCN) [89]. Moreover, a pre-clinical study has suggested that the expression of mutant p53 is essential to maintain the pro-metastatic phenotype [16].

SMAD4 is another tumor suppressor protein that transduces extracellular signals of transforming growth factor-beta (TGFβ) to the nucleus by acting as a transcriptional regulator. The major function of SMAD4 is the inhibition of cell proliferation by inducing G1 phase cell cycle arrest. *SMAD4* has been reported to be deleted or mutated in PC. Loss of SMAD4 occurs at a later stage of disease and has been associated with tumor metastases in PC. Restoration of SMAD4 was shown to sensitize PC cells to chemotherapy [90]. Moreover, genetic mutations and diminished expression of SMAD4 are found to be independently co-related with low PC patient survival [91,92]. It has been suggested that loss of SMAD4 expression is an independent prognostic factor associated with tumor progression, epithelial-mesenchymal transition (EMT) and therapy failure [93].

Phosphatase and tensin homolog (PTEN) is yet another well-characterized tumor suppressor that negatively regulates the phosphatidylinositide 3-kinase (PI3K)-RAC-alpha serine/threonine-protein kinase (AKT)-mammalian target of rapamycin (mTOR) signaling pathway [94]. It is reported to be deleted or lost in PC. PTEN loss, along with mutant KRAS, induces NF-κB activation, followed by immune cell infiltration and robust stromal activation. Further, the preclinical spontaneous mouse model of PC suggests that PTEN deficiency, along with oncogenic KRAS, exhibits the pro-metastatic potential of tumor cells [95]. Another similar study has confirmed the tumor suppressive role of PTEN in mice with the KRAS mutation [96]. A study conducted by Wartenberg and co-workers observed that the deletion of *PTEN* correlates with metastases and reduction in the overall survival of PC patients [97]. This study further suggested that tumor-associated stromal cells are deficient in PTEN protein due to chromosomal abnormality or deletion of *PTEN*, and such a defective stroma fuels pancreatic tumor cells and enhances the aggressiveness of disease. 

The proteins coded by the *BRCA1/2* genes, i.e., BRCA1 and 2, have a wide range of biological functions, including transcription regulation, DNA repair, etc. It has been shown that individuals carrying BRCA1 and BRCA2 mutations have ~2.2- and ~3.5-fold, respectively, higher risk of developing PC [98,99,100]. Additionally, Goggins and coworkers studied the frequency of biallelic inactivation of BRCA2 in the different stage (from PanIN to invasive pancreatic ductal carcinomas) of PC and reported that the wild-type allele of BRCA2 was lost in high-grade PanIN (PanIN-3), whereas no inactivation was observed in low-grade PanIN (PanIN-1) lesions [101]. Thus, their findings clearly suggest that biallelic inactivation of the *BRCA2* gene occurs only at the late stage of pancreatic tumorigenesis. The gene product of partner and localizer of BRCA2 (PALB2) works with the BRCA2 protein to repair damaged DNA by stabilizing BRAC2. Recently, it has been shown that patients with familial pancreatic cancer have mutations in their *PALB2* gene [102,103]. Mutations in the *PALB2* gene, along with other germline mutations, such as *BRAC2*, ataxia-telangiectasia mutated (*ATM*), etc., collectively alter the DNA repair pathway, resulting in increased accumulation of damaged DNA with eventual onset of cancer [104].

Individuals with hereditary pancreatitis have a higher risk of developing PC in their lifetime. Germline mutations in the protease serine 1 (*PRSS1*) gene are associated with hereditary pancreatitis [104]. The *PRSS1* gene encodes for cationic trypsinogen, abundantly present in pancreatic juice. Mutations in the *PRSS1* gene increase the conversion of trypsinogen to trypsin, and increased trypsin activity disturbs protease and anti-protease balance in the pancreas, thereby inducing pancreatitis [105,106]. A cohort study of 246 hereditary pancreatitis patients suggested an ~50-fold higher lifetime risk of developing PC in these individuals [107]. Mutations in another set of genes, the serine protease inhibitors of the Kazal type (*SPINK*), chymotrypsin C (*CTRC*) and cystic fibrosis transmembrane receptor (*CFTR*), are another risk factor for developing PC [104].

### 3.3. Epigenetic Regulation

The role of epigenetic events in the onset, as well as progression of human cancers is increasingly being realized [108]. Epigenetic regulation can explain the observed silencing of tumor suppressor genes, as well as the activation of oncogenes [109]. The knowledge of the contribution of several epigenetic events to PC progression is emerging [110]. These events include methylation, acetylation and regulation through non-coding RNAs (ncRNAs) [110,111]. It has been advocated that epigenetic changes can be pursued as possible biomarkers for early detection of PC [112] and targets for therapy [110,113]. The role of ncRNAs in PC tumorigenesis is summarized in Table 1.

ncRNAs represent a class of RNA molecules that do not encode for protein products and are now widely known for their ability to regulate a number of genes [136,137,138]. ncRNAs are also suggested to play an important role in several physiological conditions, including nearly all types of cancer [138,139,140]. The long non-coding RNA (lncRNA) H19 has been identified to be markedly overexpressed in pancreatic tumor tissues and cell lines, with a positive correlation with the invasive and migratory potential of the tumors [114,141,142]. H19 has been observed to repress the inhibitory activity of let-7 on High-mobility group AT-hook 2 (HMGA2)-mediated EMT [114]. Studies with ncRNA HOTAIR have demonstrated its overexpression in pancreatic tumor tissues, compared to adjacent non-cancerous pancreatic tissue [115]. Inhibition of HOTAIR by RNA interference (RNAi) technology in PC cell lines was observed to decrease cell proliferation, induce apoptosis and inhibit in vitro, as well as in vivo cell invasiveness [115,136]. Another ncRNA, HOTTIP, was observed to be significantly upregulated in PC tissues and cell lines. Similar to previous studies, the inhibition of HOTTIP induced proliferation arrest, impaired EMT, decreased invasion and metastasis and increased chemosensitivity to gemcitabine [116,117]. lncRNA MALAT-1 influences the growth and proliferation of PC cells [118,119,143]. Several other ncRNAs, such as AF339813, ENST00000480739, HULC (highly upregulated in liver cancer) and NUF2 (Ndc80 kinetochore complex component), have been observed to be upregulated in PC cells with profound effects on the growth of these cells [120,121,144].

In addition to the long non-coding RNAs, several other classes of ncRNAs are being routinely studied, the most common of which are the miRNAs [145]. miRNAs are 22-nucleotides in length and regulate gene expression at the post-transcriptional level by degradation of the target transcript or repression of its translation [136,139,140]. Numerous reports have indicated a role of miRNAs in PC initiation, promotion, metastasis and chemoresistance [122,123,124,125,126,146]. miR-34 is a prominent miRNA shown to be significantly downregulated in PC, and the ectopic expression of miR-34 has been demonstrated to inhibit various processes important for cancer progression [127]. Restoration of miR-96 has been shown to result in the inhibition of KRAS, thereby inducing PC cell death [128]. Similarly, inhibition of miR-21 and miR-210 decreased the invasive and metastatic potential of PC cells [147,148]. On the contrary, forced expression of miR-145 inhibited the proliferation of PC cells [149]. Several miRNAs have also been demonstrated to regulate cell-cycle-related proteins, for example regulation of cyclin-dependent kinase CDC25B by miR-148a [132], CDK6 by miR-107 [150] and CDKN1B by miR-221 [151]. We have reported suppression of MUC4 by tumor suppressor miR-150 in PC, resulting in reduced growth and tumorigenicity [133]. Further, overexpression of let-7 in PC cell lines was reported to inhibit RRM2 levels and induce chemosensitization [134]. Increased miR-211 expression in PC cells also enhanced the therapeutic efficacy by reducing ribonucleoside diphosphate reductase subunit M2 (RRM2) levels [135]. Our recent findings indicate that miR-155 induces chemoresistance in pancreatic cancer cells via inhibiting deoxycytidine kinase (DCK) expression [42]. Conversely, an upregulation of miR-146, with the downregulation of miR-205 and let-7, seems to be relevant to gemcitabine resistance in PC cells [152]. 

## 4. Deregulation of Signaling Pathways in Pancreatic Adenocarcinoma 

A growing body of evidence suggests that modulation of a single molecule is not enough for the development of human cancers, including PC. Accumulation of multiple genetic mutations causes activation of oncogenes and repression of tumor suppressor genes, resulting in activation of oncogenic signaling pathways through deregulated receptor-ligand systems (Table 2).

Upregulation of epidermal growth factor (EGF) and its receptor (EGFR) has been demonstrated to correlate with enhanced PC tumor aggressiveness and shorter survival periods [153,167,168]. Moreover, nerve-growth factor, platelet-derived growth factor, fibroblast growth factor and insulin-like growth factor, along with their receptors, exhibit elevated expression and are associated with malignant PC phenotypes [169,170,171,172]. 

Pancreatic cancer has been reported to overexpress all three isoforms of TGFβ, with an observed poor prognosis [173]. PC cells have a high expression of TGFβ receptor, TβRII, compared to normal pancreatic cells, but they are still resistant to TGFβ-induced inhibition of cell growth [174,175]. This has been observed largely due to mutations in the *Smad 4*/*DPC4* gene, a known mediator of TGFβ’s inhibitory effects [176,177]. Thus, TGFβ overexpression leads to the promotion of malignant PC through multiple mechanisms, including enhanced EMT, increased cell survival, matrix-remodeling, angiogenesis, activation of stellate cells and attenuation of immune response [178,179,180]. Expression of cytokines has also been observed to correlate negatively with cachexia and clinical outcome in PC patients [181,182,183,184]. Interestingly, different studies have reported different expression levels of certain cytokines in PC patients. This could largely be because of the method of detection employed or due to inherent differences in the populations of PC patients. Pro-inflammatory cytokines, interleukin (IL)-6, IL-8, tumor necrosis factor-α (TNF-α), IL-12, IL-18 and IL-1β, and the anti-inflammatory cytokine IL-10 have been demonstrated to be highly expressed in PC patients, compared to healthy individuals [4,164,165,185,186,187]. Engagement of the IL-6 receptor on cancer cells leads to the activation of signal transducer and activator of transcription (STATs), MAPK and PI3K, which are known to be involved in the regulation of the proliferation, survival and tumorigenicity of pancreatic cancer [188,189,190]. IL-6 has also been shown to regulate tumor angiogenesis and vascularization by controlling the secretion of vascular endothelial growth factor (VEGF) [191]. Similarly, our investigations have revealed that the secretion of IL-8 from PC cells can activate HUVEC cells; tumor cell-derived IL-8 induces endothelial cell proliferation, migration and invasion, triggering angiogenesis and providing an escape from chemotherapy [4]. IL-8 can also influence matrix remodeling through the regulation of matrix metalloproteinase-2(MMP-2), resulting in enhanced tumor invasiveness [192,193,194]. Higher levels of TNF-α, IL-8 and IL-6 have been reported in the sera of PC patients, compared to healthy controls, suggesting their role in PC pathogenesis [195]. Furthermore, elevated levels ofIL-6 are associated with poor PC patient survival, and there is preclinical evidence to suggest that IL-6 is required for the development and progression of pancreatic tumor precursor lesions [196,197]. In recent years, our own studies have established the involvement of stromal-derived factor (SDF-1)/CXCL12 and the CXCL12-CXCR4 signaling in PC tumorigenesis and chemoresistance [26,198]. We have also reported that an unintended consequence of chemotherapy in PC is the upregulation of CXCR4 receptors, which make pancreatic tumor cells highly aggressive [27]. 

While controlling inflammatory responses in different types of cells, NF-κB signaling is also involved in the control of development, apoptosis and cell proliferation [199]. The NF-κB factors involve RelA (p65), RelB, c-Rel, p105/p50 and p100/p52 that are present in the cytoplasm as homo- or hetero-dimers and kept in an inactive state by the inhibitor of kappa B- alpha (IκB-α) protein. With an appropriate signal, IκB-α is phosphorylated and degraded, leading to the release, activation and nuclear localization of NF-κB, which results in the expression of its target genes [200]. As inflammatory cues have been demonstrated to induce PC progression and are also responsible for the activation of NF-κB signaling, a direct causal relation between the two has been well established. In fact, not only does the NF-κB signaling work towards the development/progression of cancer, it helps in the maintenance of the tumor cells, the initiation of metastatic cascades by the direct regulation of MMPs, the induction of angiogenesis through the VEGF signaling in the micro-environment and tissue invasion at distal organs [162,163,201]. We have also demonstrated a significant role of NF-κB signaling in the development of gemcitabine resistance in pancreatic cancer through ROS-mediated activation of NF-κB and the subsequent upregulation of CXCR4-signaling, thereby promoting cell survival [27].

Secreted mucins are the main component of mucus that protects epithelial cells; they also play important biological roles in cell-cell/cell-matrix interactions and tumor cell signaling. Altered expression of mucins in PC has been reported earlier [202,203]. Membrane-bound mucins contain a transmembrane domain, and there are almost 20 identified MUCs, such as MUC1, MUC3, MUC4, MUC12, MUC13, MUC15, MUC16, MUC17, MUC20 and MUC21. Among them, MUC1 and MUC4 are best characterized in pancreatic tumorigenesis. Overexpression of MUC1 was observed in PanIN-3 and invasive ductal carcinoma [204]. Downregulation of MUC1 expression, using RNA interference, has been shown to decrease the metastatic potential of pancreatic adenocarcinoma cells. MUC4 has been reported to activate Akt and MAPK pathways, leading to the induction of cell proliferation and survival. MUC4 expression gradually increases during carcinogenesis, as evidenced by immunohistochemical analysis: 17% of PanIN-1A, 36% of PanIN-2 and 85% of PanIN-3 express MUC4; in PDAC, the prevalence of MUC4 is ~83% to 89% [205,206,207]. Further, we have demonstrated that ectopic expression of miR-150, which is frequently downregulated in PC, in MUC4-expressing cell lines reduces MUC4 expression and abolishes MUC4-dependent PC pathogenesis [133].

Hedgehog (Hh) signaling is involved in embryonic development and regulates multiple cellular processes. Humans have three hedgehog homologs; DHH (desert hedgehog), IHH (Indian hedgehog) and SHH (Sonic hedgehog). These molecules serve as the Hh ligands [208]. The hedgehog pathway becomes functionally active when ligand binds to its transmembrane patched receptor (Ptch), inducing its internalization and the release of smoothened (Smo). Activated Smo leads to the migration of Gli transcription factor from cytosol to the nucleus, which executes the expression of Gli-dependent genes (Cyclin D, Myc, Gli1, Ptch, etc.). Hh signaling is constitutively active in many human cancers including PC [208,209]. Hh signaling was reported to be active in several PC cell lines, as well as in PC patient-derived samples [210]. The development of desmoplasia in the pancreatic tumor has been reported to be mediated through the activation of the Hh signaling pathway by the stroma-derived SHH [211]. Such a dense desmoplastic reaction is considered the cornerstone of increased chemoresistance due to reduced tumor vasculature and decreased drug accumulation at the tumor site [28]. However, the inhibition of SHH secretion in the pancreatic tumors failed to provide clinical benefit to the therapy of pancreatic cancer; it, rather, led to increased tumor metastasis [212].

The NOTCH pathway, similar to Hh signaling, is mostly active during embryogenesis. It is also reported to be activated in many human cancers, including PC [213,214,215,216]. Activation of NOTCH signaling requires the binding of NOTCH ligand directly to the receptor on the cell membrane, and the effects of NOTCH signaling on the tumor compartment are far better characterized, as compared to its impact on the stroma. In cancer cells, NOTCH functions to maintain a steady low level of an undifferentiated population of cells that serve as the cancer stem cells [216,217]. In fact, overexpression and activation of NOTCH signaling has been shown to increase in early PanIN lesions, compared to normal pancreas [156]. Additionally, an upregulation of NOTCH has also been demonstrated to be required for pancreatic cancer tumor maintenance, as observed through its upregulation in the *Pdx1-Cre*; *Kras^G12D^*; *p53^lox/+^* mouse model and the upregulation of NOTCH pathways in resulting mouse lesions [157,218]. Interestingly, inhibition of γ-secretase, the enzyme responsible for the cleavage of the NOTCH receptor, in this mouse model resulted in significant inhibition of the development of invasive carcinoma [219]. The NOTCH pathway has also been demonstrated to crosstalk with several other oncogenic signaling pathways, such as the NF-κB signaling and the EGFR pathway [178,220]. EGFR signaling has been shown to activate NOTCH, and inhibition of NOTCH has been observed to decrease the transactivation of NF-κB signaling [221,222]. Moreover, downregulation of NOTCH signaling results in reduced aggressiveness of pancreatic cancer cells [223]. 

WNT (Wingless/Integrated) signaling is also known for its role in cell growth and differentiation during development, and it has been reported that alterations in WNT signaling induce changes in the tumor-compartment of PC [160]. Binding of WNT ligand to its receptor leads to the release of β-catenin in the cytoplasm and translocation to nucleus, where it associates with the transcription factor, T-cell factor/lymphoid enhancer-binding factor (TCF-LEF) to activate its target genes [224]. Pancreatic cancer, similar to other cancers, has been reported to overexpress β-catenin, compared to normal tissues [224,225,226]. The regenerative microenvironment of acinar cells, following injury, mimics pancreatitis and is characterized by transient reactivation of embryonic-development pathways, such as WNT/β-catenin [227]. Interestingly, activated β-catenin signaling has been observed to antagonize KRAS-induced transformation; but the inflammatory state provides a break for constitutively-active KRAS to induce early events in pancreatic ductal adenocarcinoma initiation by blocking acinar regeneration. Genetic and chemical ablation of β-catenin in pancreatic acinar cells has been shown to significantly delay PanIN formation [160], and the forced overexpression of constitutively-active, degradation-resistant β-catenin, combined with mutant KRAS, has been shown to result in a rare form of pancreatic tumors, reminiscent of human intraductal tubular tumors (ITT), unrelated to PDACs [228]. A critical level of β-catenin activity is required for KRAS-induced acinar-to-ductal reprogramming in PanIN [228].

## 5. Conclusions and Future Perspectives

Recent years have witnessed significant advancements in the understanding of the molecular events responsible for pancreatic cancer pathogenesis. Inactivation of tumor suppressor genes/activation of oncogenes along with deregulation of various signaling pathways have been suggested to be critical in PC pathobiology. However, despite these advances, PC remains a challenge to clinicians and researchers alike. Its early detection still remains the best bet for a successful clinical outcome. A number of identified events, both genetic, as well as epigenetic, have been evaluated in mutually-exclusive experiments in laboratory settings. These findings have often been validated in vivo in immune-/genetically-compromised mice. While this, arguably, is a logical and standard approach, more robust studies need to be urgently planned. This is particularly critical as PC continues to climb the rankings for the most lethal cancers. A good starting point could be studies focused on the tumor microenvironment. This is based on the evidence from recent literature suggesting the significance of stroma in pancreatic cancer pathobiology. In consideration of the conflicting reports on the role of desmoplasia (whether it provides sanctuary to the growing pancreatic tumors and protects them from chemotherapy vs. whether it functions to restrain tumor growth), it has become even more important to evaluate the exact contribution of the desmoplastic tumor microenvironment and its associated factors in PC pathogenesis and chemoresistance. Such information will directly impact the chemotherapeutic management of PC. Towards this goal, use of patient-derived xenograft (PDX) and organoid models is important in future mechanistic studies and will help unravel many mysteries associated with complex PC pathobiology. It is evident that focusing on individual factors for their role in PC pathogenesis for putative targeted therapy might not be a very effective strategy in our fight against PC. The more complex interplay of several factors needs to be appreciated and evaluated using appropriate model systems to advance PC research.

## Figures and Tables

**Table 1:** Non-coding RNAs in pancreatic cancer (PC).

Non-Coding RNAs	Molecular Targets	Role in Pancreatic Cancer	References
H19	HMGA2, let-7	EMT	[114]
HOTAIR	Genes associated to cell cycle	proliferation, Invasion	[115]
HOTTIP	HOX genes	survival, proliferation, migration	[116,117]
MALAT1	Genes associated with cell cycle and EMT	cell proliferation, migration invasion, prognostic marker	[118,119]
AF339813	NUF2	cell proliferation, apoptosis	[120]
ENST00000480739	OS-9 and HIF-1α	invasion	[121]
miR-367	SMAD7	EMT, invasion	[122]
miR-29c	MMP2	metastasis	[123]
miR-23a	APAF1	cell proliferation and apoptosis	[124]
miR-223	Fbw7	EMT	[125]
miR-206	ANXA2 and KRAS	cell proliferation, invasion and lymphangiogenesis	[126]
miR-34	Bcl-2 and NOTCH	maintenance and survival of cancer stem cells	[127]
miR-96	KRAS	tumor cell growth, invasion and migration	[128]
miR-21	MMP-2, MMP-9	metastasis	[129]
miR-210	vimentin and snai-1	invasion, migration	[130]
miR-145	MUC13	PC cell growth and invasion	[131]
miR-148a	CDC25B	PC cell survival	[132]
miR-150	MUC4	growth migration and invasion	[133]
let-7, miR-211	RRM2	chemoresistance	[134,135]
miR-155	DCK	chemoresistance	[42]

HMGA2: High-mobility group AT-hook 2; HOX: Homeobox; EMT: epithelial-mesenchymal transition; NUF2: Ndc80 kinetochore complex component; OS-9: Osteosarcoma amplified 9; HIF-1α: Hypoxia inducible factor-1 alpha; SMAD7: Mothers against decapentaplegic homolog 7; MMP: Matrix metalloproteinase; APAF1: Apoptotic protease activating factor 1; Fbw7: F-box/wd repeat-containing protein 7; ANXA2: Annexin A2; MUC13: Mucin 13; CDC25B: Cell division cycle 25B; RRM2: Ribonucleoside diphosphate reductase subunit M2; DCK: deoxycytidine kinase.

**Table 2:** Altered signaling pathways in pancreatic malignancy.

Signaling Pathway	Signaling Molecules Involved in PC Tumorigenesis	References
EGFR signaling	HER2/neu, PI3K, Akt, ERK, Ras/Raf, TGF-α	[153,154,155]
NOTCH signaling	γ-secretase, JAGGED2, DLL3/4	[156,157]
Hedgehog signaling	SHH, Gli, PTCH, Smo, PI3K/AKT, MMPs	[158,159]
WNT signaling	β-catenin, TCF/LEF, MAPK, Dkk1, GSK3β	[160,161]
NF-κB signaling	Bcl-xL, Bcl-2, SHH, CXCR4, MMPs, VEGF	[27,162,163]
Cytokines/growth factors associated signaling	PDGF, TGF-β, CXCL12, IL-6, TNF-α, IL-8, IL-12, IL-18, IL-1β, IL-10, STATs, MAPK, PI3K, MMPs, CCL28	[26,164,165,166]

Akt: RAC-alpha serine/threonine-protein kinase; CCL28: C-C motif chemokine ligand 28; CXCL12: C-X-C motif chemokine ligand 12; CXCR4: C-X-C motif chemokine receptor 4; Dkk1: Dickkopf WNT signaling pathway inhibitor 1; DLL3/4: Delta like canonical Notch ligand 3/4; EGFR: Epidermal growth factor receptor; ERK: Extracellular regulated MAP kinase; GSK3β: Glycogen synthase kinase 3 beta; HER2/neu: Human epidermal growth factor receptor 2; IL: Interleukin; MAPK: Mitogen activated protein kinase; MMPs: Matrix metalloproteinases; NF-κB: Nuclear factor-kappa B; PDGF: Platelet derived growth factor; PI3K: Phosphatidylinositide 3-kinase; PTCH: Patched; Ras: rat sarcoma oncogene homolog; Raf: Rapidly accelerated fibrosarcoma; SHH: Sonic hedgehog; Smo: Smoothened; STAT: Signal transducer and activator of transcription; TCF: Transcription factor; LEF: Lymphoid enhancer-binding factor 1; TGF: transforming growth factor; VEGF: Vascular endothelial growth factor; WNT: Wingless/Integrated

## Abbreviations

ADM	adrenomedullin
APC	adenomatous polyposis coli
CAFs	cancer-associated fibroblasts
CDK	cyclin-dependent kinase
CFTR	cystic fibrosis transmembrane receptor
CTRC	chymotrypsin c
DHH	desert hedgehog
DPC4	deleted in pancreatic cancer, locus 4
ECM	extra cellular matrix
EGF	epidermal growth factor
EGFR	epidermal growth factor receptor
GPC1	glypican-1
HA	hyaluronan
HER2	human epidermal growth factor receptor 2
Hh	hedgehog
HULC	highly upregulated in liver cancer
IHH	Indian hedgehog
lncRNA	long non-coding RNA
MCN	mucinous cystic neoplasm
miRNAs	microRNAs
MPCS	melanoma pancreatic cancer syndrome
ncRNAs	non-coding RNAs
PAK4	p21-activated kinase 4
PanIN	pancreatic intraepithelial neoplasia
PanNET	pancreatic neuroendocrine tumor
PC	pancreatic cancer
PDAC	pancreatic ductal adenocarcinoma
SDF-1	stromal-derived factor 1
SHH	Sonic hedgehog
Smo	smoothened
SOCS1	suppressor of cytokine signaling 1
TGFβ	transforming growth factor-beta
TIMP1	tissue inhibitor of metalloproteinases-1
TME	tumor micro-environment
